# Prepregnancy Endocrine, Autoimmune Disorders and the Risks of Gestational Hypertension-Preeclampsia in Primiparas: A Nationwide Population-Based Study in Taiwan

**DOI:** 10.3390/ijerph17103657

**Published:** 2020-05-22

**Authors:** Mei-Lien Pan, Li-Ru Chen, Hsiao-Mei Tsao, Kuo-Hu Chen

**Affiliations:** 1 Institute of Information Science, Academia Sinica, Taipei 115, Taiwan; mlpan66@gmail.com (M.-L.P.); hsiaomei.tsao@gmail.com (H.-M.T.); 2 Department of Physical Medicine and Rehabilitation, Mackay Memorial Hospital, Taipei 104, Taiwan; gracealex168@gmail.com; 3 Department of Mechanical Engineering, National Chiao-Tung University, Hsinchu 300, Taiwan; 4 Department of Obstetrics and Gynecology, Taipei Tzu-Chi Hospital, Buddhist Tzu-Chi Medical Foundation, Taipei 231, Taiwan; 5 School of Medicine, Tzu-Chi University, Hualien 970, Taiwan

**Keywords:** gestational hypertension, preeclampsia, polycystic ovarian syndrome (PCOS), systemic lupus erythematosus (SLE)

## Abstract

(1) Objective: To assess the risks of gestational hypertension/preeclampsia (GH-PE) in women with prepregnancy endocrine and autoimmune disorders such as polycystic ovarian syndrome (PCOS) and systemic lupus erythematosus (SLE). (2) Methods: In a nationwide population-based longitudinal study, data were retrieved from the 1998 to 2012 Taiwan National Health Insurance Research Database. ICD9-CM codes 256.4, 710.0, and 642.X were identified for the corresponding diagnoses of PCOS, SLE, and GH-PE, respectively, which were further confirmed by inspection of medical claims data for ultrasonography findings, laboratory tests, blood pressure measurements and examinations of urine protein to ensure the accuracy of the diagnoses. To clarify the risks of primiparous GH-PE, the study excluded women diagnosed with PCOS or SLE at <15 or >45 years of age, pre-existing chronic hypertension, GH-PE before PCOS and SLE, and abortion or termination before 20 weeks’ gestation. For women affected by prepregnancy PCOS or SLE individually, each pregnant woman was age-matched to four pregnant women without PCOS or SLE. Logistic regression analyses were applied to report odds ratios (ORs) for the risks of GH-PE after adjustment for age, occupation, urbanization, economic status, and other co-morbidities. (3) Results: Among 8070 and 2430 women with prepregnancy PCOS and SLE retrieved from a population of 1,000,000 residents, 1953 (24.20%) and 820 (33.74%) had subsequent primiparous pregnancies that were analyzable and compared with 7812 and 3280 pregnancies without prepregnancy PCOS and SLE, respectively. GH-PE occurred more frequently in pregnancies with prepregnancy PCOS (5.79% vs. 2.23%, *p* < 0.0001) and SLE (3.41% vs. 1.80%, *p* < 0.01) as compared to those without PCOS and SLE. Further analysis revealed that prepregnancy PCOS (adjusted OR = 2.36; 95%CI: 1.83–3.05) and SLE (adjusted OR = 1.95; 95%CI: 1.23–3.10) were individually associated with GH-PE. The risk of GH-PE was not reduced in women with prepregnancy PCOS receiving metformin treatment (*p =* 0.22). (4) Conclusions: Prepregnancy PCOS and SLE are independent and significant risk factors for the occurrence of GH-PE. Because the peripartum complications are much higher among pregnancies with GH-PE, the at-risk woman should be informed and well-prepared during her pregnancy and delivery.

## 1. Introduction

Systemic lupus erythematosus (SLE) and polycystic ovary syndrome (PCOS) are two common disorders which may affect subsequent pregnancies among women with prepregnancy autoimmune and endocrine disorders. SLE is a multifactorial autoimmune disease, with evidence of genetic susceptibility, environmental effects, and disturbances in both innate and adaptive immunity, manifested by disturbances in apoptotic cell clearance, cytokines, B-cell immunity, and T-cell signaling [1]. It is defined by the following clinical criteria: acute or chronic cutaneous lupus, oral or nasal ulcers, synovitis or serositis, renal or neurological involvement, hemolytic anemia, and leukopenia or thrombocytopenia. Immunological criteria include elevated anti-nuclear antibodies, anti-double-stranded-DNA antibodies, anti-phospholipid antibodies, and low complement C3 and C4 [1,2]. Microscopically, lupus is strongly associated with defects in apoptotic clearance [1,2], and the resulting excessive apoptotic debris has an important pathogenic effect. These apoptotic cells express signals to attract macrophages and dendritic cells, thus inducing immunologic reactions. The inability of phagocytes to remove apoptotic material leads to exposure of nuclear particle fragments to antigen-presenting cells, subsequent interactions with T and B cells, and eventual development of antinuclear antibodies [1]. On the other hand, PCOS, according to the Rotterdam criteria [3], is an endocrine disorder resulting from ovarian dysfunction that is characterized by chronic anovulation, hyperandrogenism, and special morphologic changes of the ovaries under ultrasonographic examination [4,5,6,7]. The prevalence of PCOS is approximately 5% to 14% among reproductive-aged women [5,6], and affected patients generally present with symptoms and signs of menstrual irregularity, infertility, obesity, and androgen excess [7]. As many as half of the women with PCOS have co-existing metabolic syndrome [8,9], among whom insulin resistance is common, with the risk of developing type 2 diabetes mellitus (DM) five- to eight-fold higher than women without PCOS [9,10]. Associated immunological disorders such as elevated levels of autoimmune antibodies [11,12,13,14] and cytokines [15,16] are more common among PCOS patients. Although the etiology of PCOS remains unclear, current evidence suggests that the possible pathophysiology of PCOS is gene-related insulin resistance, which induces the consequent hyperinsulinemia, and stimulates excessive ovarian androgen production as well as blocking follicular maturation [16,17,18,19].

Hypertension is the most common medical complication during pregnancy. Approximately 70% of hypertension during pregnancy can be attributed to gestational hypertension/preeclampsia (GH-PE), which has been reported to have a prevalence of 6% to 8% in pregnant women [20]. The definition of GH is a higher systolic (≥140 mmHg) or diastolic (≥90 mmHg) blood pressure after 20 weeks without the finding of urinary protein [20,21]. Among 2% to 5% of all pregnancies, GH will progress to PE, defined as the presence of elevated BP (≥140/90 mmHg), proteinuria or the dysfunction of multiple organs [20,21,22,23,24]. As a major cause of fetal and maternal deaths, [21,22,24], PE also predisposes fetuses and mothers to subsequent cardiovascular diseases [22,23,25,26,27]. The risk factors of GH-PE include first pregnancy, multifetal pregnancy, prepregnancy obesity or higher body-mass-index (BMI), old age, and overt diabetes [20,21,22,23,28,29,30,31,32,33,34]. We have found that preterm placental calcification is a major risk factor for progression to PE [35], adverse maternal and neonatal outcomes [36,37], and even stillbirth [38]. Nowadays, both the etiology and pathophysiology underlying GH-PE is seen as a cascade of events including abnormal trophoblastic invasion during placentation, subsequent ischemia of placentas and injuries of endothelium [20,21,23,28,39,40].

Even though the association of PCOS with infertility is now very clear, there are few studies exploring the connection between PCOS or SLE and GH-PE. Research drawn from the Ovid-Medline database revealed contradicting conclusions. Some studies reported that preexisting PCOS [41,42,43,44] and SLE [45,46,47] increased the risk of subsequent GH-PE, while other studies reported no connection between PCOS and GH-PE [48,49]. It is confusing that these aforementioned studies with different samples and research designs revealed conflicting conclusions. The major limitations of previous studies on the association of PCOS or SLE with GH-PE were retrospective design and smaller sample size [42,43,44,46,47,49], making the conclusions less reliable. Two studies with larger sample sizes were performed using physician-identified inclusion criteria [41,45] rather than ultrasonographic and laboratory examinations for confirmation of PCOS, SLE, and GH-PE. Without strict standards for diagnoses, subjective recognition by the physician may lead to selection bias and a substantial confounding effect on their results. It is therefore important to investigate the effect of prepregnancy PCOS and SLE on subsequent GH-PE by analyzing a large nationwide population with stricter selection criteria to reach convincing conclusions.

## 2. Materials and Methods

### 2.1. Data Source and Sample

Launched in 1995, the National Health Insurance (NHI) is a single-payer health insurance program, which covered 93% of the population in Taiwan by 1997, with coverage increased to 99% in 2011. The NHI program uses International Classification of Diseases, 9th Revision, Clinical Modification (ICD-9-CM) codes for disease diagnosis and NHI-specific codes for procedures or treatments. Extracted from the claims data of the NHI program, the NHI Research Database (NHIRD) contains nationwide information on outpatient and inpatient medical claims including dates of clinical visits, demographic data, and records of disease diagnosis and treatment of anonymous insured patients. All data in the NHIRD are de-linked before release, by means of deleting the identification codes of healthcare providers and patients. The Longitudinal Health Insurance Database (LHID) 2010 is a longitudinal cohort data subset in 2010 of the NHIRD, and it consists of the claims data of 1,000,000 randomly sampled individuals insured in 2010. Compared with the entire population in the NHI program, the LHID2010 subset has no significant difference in age, sex, or healthcare costs. The data in the current study were retrieved from the LHID2010 subset of NHIRD, and we enrolled eligible individuals from the nationwide population-based database rather than recruiting participants after obtaining informed consent. Thus, there was no participant who could “participate in” or “withdraw from” the study. Our previous experience of a population-based study using LHID [50] revealed that the major causes of data censoring were individual withdrawal from the NHI and death.

### 2.2. Statement of Research Ethics

Since all claims data in the NHIRD were anonymized and de-linked before release, the requirement for informed consent was waived according to the local regulations. This study was granted for exempt review (protocol number: 08-W-056) by the institutional review board of Taipei TzuChi hospital, Taiwan.

### 2.3. Inclusion and Exclusion Criteria

From the LHID2010 subset between 1998 and 2012, women previously diagnosed with PCOS (ICD-9-CM code 256.4) or SLE (ICD-9-CM code 710.0) were selected and designated as the exposure groups after they became pregnant. Initially, basic screening was arranged for exclusion of women with missing data. Women with inconsistent diagnoses of PCOS, SLE, or GH-PE (ICD-9-CM codes 642.X) in personal records of the national database, indicating discrepancies of ICD codes or outpatient and inpatient medical claims data, were also excluded. The date of the first valid diagnosis of PCOS or SLE was defined as the index date.

Although there was a lack of worldwide consensus regarding the diagnosis of PCOS until 2003 when the Rotterdam criteria were established [3], the diagnosis of PCOS was effectively made in Taiwan if physicians noted more than one of the following changes: ovarian dysfunction; hyperandrogenism; or polycystic ovary morphology. Basing the diagnosis on symptoms, ultrasonographic findings, and laboratory test results makes coding for PCOS stricter and more reliable. Therefore, the diagnosis of PCOS was not considered valid unless personal records of medical claims in the national database included blood tests for LH, FSH, or testosterone (NHI codes: 09078B2, 09126B, 09126C, 09078B1, 09125B, 09125C, 09064B2, 09121B, and 09121C) and gynecologic ultrasonography (NHI code: 19003C). For confirmation, we reviewed the blood test results and gynecologic ultrasonographic findings, which had been obtained within 90 days before a diagnosis of PCOS was made. For all of the affected women, the diagnosis of PCOS was established based on objective changes in blood hormones and findings on ultrasonography. Similarly, the valid diagnosis of SLE was assured based on reviewing personal records of medical claims in the national database, including anti-nuclear antibodies, anti-double-stranded-DNA antibodies, anti-phospholipid antibodies, and complement C3 and C4 (NHI codes: 12053C, 12060B, 30027B, 12034B, and 12038B).

For clarification of the future GH-PE risk among reproductive-aged women, those aged <15 or >45 were excluded. Women with pre-existing chronic hypertension (401.X-405.X) were excluded because the main focus of the study was GH-PE. Furthermore, women with GH-PE prior to PCOS or SLE were also excluded. Because antenatal visits are covered in the NHI program, nearly all pregnant women had regular antenatal visits. When screening the national database, the diagnosis was considered invalid if there was a lack of medical claims data including antenatal visit records or reports of urine protein and blood pressure among women diagnosed with GH-PE. As the diagnostic criteria of PCOS, SLE, and GH-PE for this study were based on reviews of medical claims in the database, the diagnoses and coding of PCOS, SLE, and GH-PE were strict and reliable.

**Figure 1:**
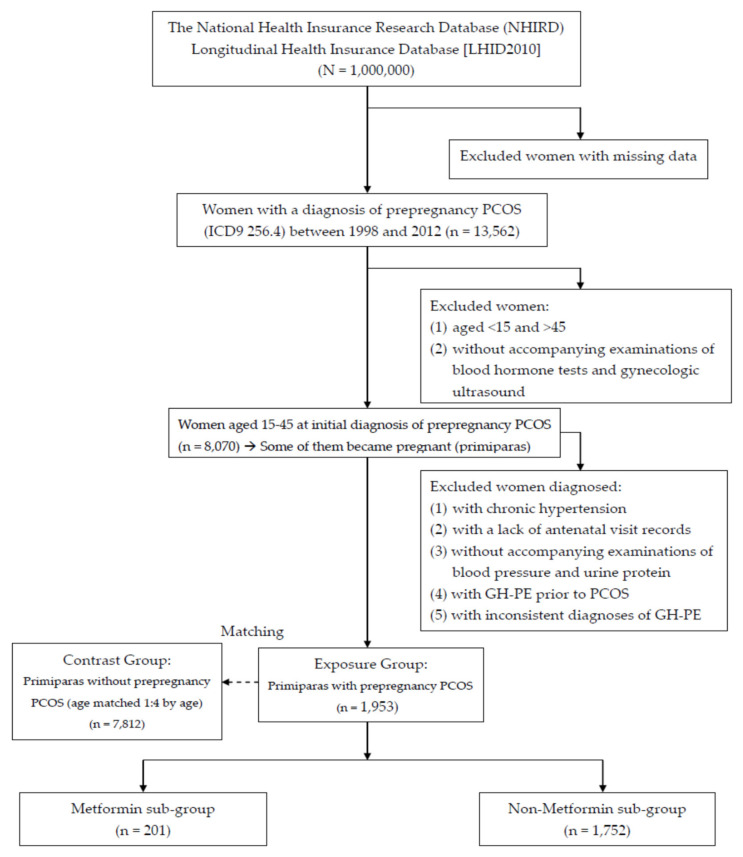
The flow chart of case inclusion and exclusion of women with prepregnancy polycystic ovary syndrome (PCOS).

**Figure 2:**
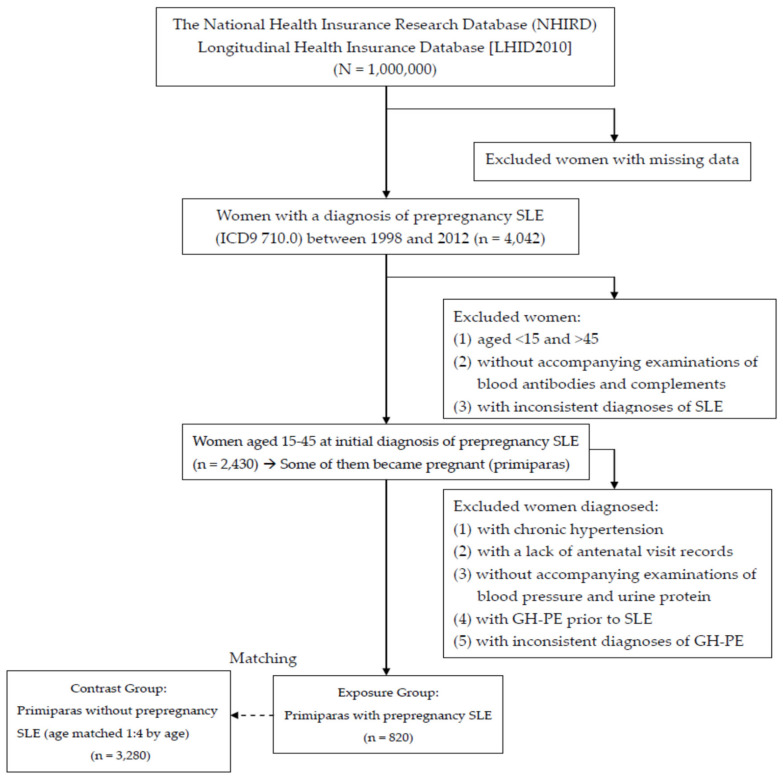
The flow chart of case inclusion and exclusion of women with prepregnancy systemic lupus erythematosus (SLE).

Because this study excluded women who were aged <15 or >45 and those with inconsistent diagnoses to ensure the accuracy of the diagnoses, the women without PCOS or SLE were selected from the remaining women in the LHID2010 and identified as the contrast group. During pregnancy, every woman with prepregnancy PCOS or SLE (exposure group) was matched with four women of the same age without prepregnancy PCOS or SLE (contrast group). Each woman in the contrast group was assigned an index date identical to that of the corresponding exposure group. All pregnancies in the exposure and contrast groups were then identified and examined for the occurrence of GH-PE. If a woman had many pregnancies complicated with GH-PE, only the first GH-PE was analyzed. Because parity is a risk factor of GH-PE, we analyzed the first pregnancy at which GH-PE had occurred in the exposure and contrast groups. Figure 1 and Figure 2 show the flowchart of case inclusion, exclusion, and classification.

In order to investigate the effect of prior PCOS on future GH-PE, this study further explored whether the use of metformin, a common oral biguanide for treatment of PCOS, decreased the risk of GH-PE. Patients with preceding PCOS were then categorized into two subgroups according to use of metformin. The women who had taken metformin solely were defined as the metformin subgroup and compared with those without metformin usage. Metformin usage was identified for sub-analyses only if it preceded the index pregnancy.

Among women in the exposure and contrast groups, we compared the demographic characteristics, including age at first pregnancy, age at the diagnosis of PCOS or SLE, occupation, economic status, urbanization and co-morbidities. Occupation was classified as white collar, blue collar, and retired/others. Urbanization was categorized as urban, suburban, or rural. The data of economic status were not available in the NHI system, but could be substituted with insurable wage as a proxy measurement. Thus, economic status was classified as four subgroups (New Taiwan Dollar [NTD] 1.00 = US$ 0.033): insurable wage ≧NTD 40,000; NTD 20,000–40,000; <NTD 20,000; and retired/others. Co-morbidities included diabetes mellitus (250.X), dyslipidemia (272.X), ischemic heart disease (410.X-414.X), cerebrovascular disease (430.X-438.X), and chronic pulmonary disease (490.X–496.X). The current study included both previously-identified risk factors (age at diagnosis; parity of pregnancies) and confounding factors (occupation, economic status, urbanization and co-morbidities) for an initial analysis. The significant factors would be analyzed in further regression models.

### 2.4. Data Analysis

SAS (version 9.4; SAS Institute, Inc., Cary, NC, USA) was applied to data analyses. The chi square test and student’s t-test were used for categorical and continuous variables, respectively. Demographic characteristics including age at PCOS or SLE diagnosis, occupation, economic status, urbanization and co-morbidities were analyzed and compared between the exposure and contrast groups. For the risk of GH-PE, logistic regression analyses were used for reporting odds ratio (OR) and the 95% confidence interval (95% CI) after adjusting for significant factors. *P*-value < 0.05 was considered significant.

## 3. Results

The analysis of the 1,000,000 nationals insured in 2010 showed 13,562 females with PCOS and 4042 females with SLE based on ICD-9. The women with PCOS included 13,136 reproductive-aged (15~45) and 426 non-reproductive-aged females; the women with SLE included 2535 reproductive-aged (15~45) and 1507 non-reproductive-aged females. According to the criteria for case inclusion and exclusion, we identified 8070 PCOS and 2430 SLE women between 1998 and 2012 (Figure 1 and Figure 2). Among those women with prepregnancy PCOS and SLE, ,953 (24.20%) and 820 (33.74%) had subsequent primiparous pregnancies that were analyzable. Each woman with prepregnancy PCOS or SLE (exposure group) was age-matched to four pregnant women of the same age without prepregnancy PCOS or SLE (contrast group). Pregnancies in the exposure groups were compared with 7812 and 3280 age-matched pregnancies in the contrast groups, respectively. For subsequent pregnancies, comparisons of the characteristics between the exposure and contrast groups are listed in Table 1 and Table 2. The age in Table 1 and Table 2 provide the women’s age (15–25, 26–35, and 36–45 years) when PCOS and SLE were initially diagnosed.

For the exposure groups, the average age at the time of initial diagnosis of PCOS and SLE was 27.49 ± 4.83 and 29.51 ± 6.44 years. Approximately 50% to 60% of these women were first diagnosed with PCOS and SLE between age 26 and 35. For all women in the analysis, the majority in the exposure and contrast groups were white-collar workers (>50%), urban area residents (>60%), and middle-high level economic status (40%–50%). For both PCOS and SLE, there were significant differences in insurable wage between the exposure and contrast groups. Between the exposure (PCOS) and contrast (no PCOS) groups, there were significant differences in some of the comorbidities including diabetes mellitus, dyslipidemia, and cerebrovascular disease. On the other hand, there were significant differences in all of the comorbidities between exposure (SLE) and contrast (no SLE) groups (Table 1 and Table 2).

Table 3 is the risk analyses of prepregnancy PCOS or SLE. There were 113 cases of GH-PE out of 1953 women with prepregnanacy PCOS and 174 cases out of 7812 women without prepregnancy PCOS (5.79% vs. 2.23%, *p* < 0.001). Likewise, there were 28 cases of GH-PE in 820 women with prepregnanacy SLE and 59 cases in 3280 women without prepregnanacy SLE (3.41% vs. 1.80%, *p* < 0.01). Compared with women in the contrast group, those in the exposure group (PCOS; SLE) had a higher incidence of GH-PE. Logistic regression analyses demonstrated that prepregnancy PCOS or SLE is an independent and significant risk factor for GH-PE (adjusted OR: 2.36; 95% CI: 1.83–3.05 for PCOS; adjusted OR: 1.95; 95% CI: 1.23–3.10 for SLE).

We further explored whether the use of metformin for treating prepregnancy PCOS decreased the risk of GH-PE (Table 3). Among the 1953 women with prepregnancy PCOS, 201 (10.30%) had used metformin solely. Between metformin and non-metformin subgroups, the incidence of GH-PE was 7.46% (15/201) and 5.59% (98/1752), respectively. In the subgroup analysis, the use of metformin for prepregnancy PCOS did not lower the risk of future GH-PE (adjusted OR: 1.44; 95% CI: 0.81-2.56).

## 4. Discussion

The relationship between pre-existing diabetes or thyroid dysfunction and future pregnancy complications (such as PE) is well-known. In contrast, PCOS is a good focus because it affects females only, and its connection with future PE is less explored. The autoimmune disease SLE (the prevalence: female > male) is chosen as another focus because it usually worsens during pregnancy due to increased release of lupus coagulant, and its complications are often severe for pregnant women. The results of this study revealed that women with either PCOS or SLE have a higher incidence of future GH-PE compared to those without PCOS or SLE. After adjustment with covariates, prepregnancy PCOS or SLE is an independent and significant risk factor (adjusted OR: 2.36 and 1.95, respectively) for GH-PE. Women with prepregnancy PCOS or SLE are predisposed to a higher risk of developing future GH-PE, which calls for closer surveillance of both fetal and maternal well-being. After confirming previous PCOS or SLE, at-risk pregnant women should be provided with appropriate information and suggestions to facilitate specialty referral or early intervention. Before and during delivery, preparation and monitoring should be increased for affected women to avoid serious complications of GH-PE. Furthermore, the risk of future GH-PE has not been found to be significantly lowered in women with PCOS after receiving metformin treatment.

Our results revealed that prepregnancy SLE is an independent and significant risk factor for GH-PE. It is well known that lupus activity is increased at the time of conception [1], and the damage associated with SLE before and during conception may be responsible for lupus vasculitis, loss of elasticity of vessels, and subsequent hypertension. This theoretical basis is supported by the findings in other studies that abnormal levels of VEGF, PlGF, and sFlt-1 are common changes in patients with SLE, and can be predictive of preeclampsia [51,52]. Lower levels of proangiogenic factors VEGF and PlGF, and higher levels of antiangiogenic factor sFlt-1 impair angiogenesis, lead to placental endothelial dysfunction as well as poor placental perfusion, and predispose to preeclampsia [51]. In an experimental model, anti-phospholipid antibodies generated in patients with SLE target the maternal-fetal interface, activate complement, recruit and stimulate leukocytes, and increase the production of antiangiogenic factors and TNF-α. Inflammatory injury provokes the release of sFlt1 by trophoblasts. Interferon-α, a key pathogenic cytokine and potent antiangiogenic factor, suppresses endothelial cell production of VEGF and increases sFlt1 [51]. An enhanced release of sFlt1 attenuates uterine spiral artery remodeling and impairs placental perfusion, whereas interferon-α combined with sFlt1 disrupts the ability of trophoblasts to interact with endothelial cells [52,53,54,55]. Our findings provide evidence for identifying high-risk patients and a rationale for further interventions that investigate the blockage of upstream or downstream factors in the target pathways.

Similar to the results of previous reports [56,57], pregnant women with prepregnancy PCOS have a higher risk of developing GH-PE compared with women without prepregnancy PCOS. In PCOS, hyperandrogenism is closely related to the incidence and extension of microscopic alterations in early trophoblast invasion and placenta development [58]. In pregnancies with hypertensive complications, serum testosterone and sex hormone-binding globulin concentrations are significantly increased and decreased, respectively, implying that free testosterone might mediate hemodynamic changes underlying PE by inducing a state of sympathetic and vascular hyperactivity [58]. Considering the direct effect of androgens on the endometrium and/or to specific tissue susceptibility, the alterations in endovascular trophoblast invasion and placenta development may be the result of a suboptimal implantation process [58]. Another study of biomarkers between women with PCOS and PE suggested that immune regulation, inflammation, and antioxidants may play key roles in the pathophysiologic mechanisms [59]. To investigate the possible mechanisms underlying the diseases, proteomic biomarkers were identified and might clarify the link between PCOS and PE [59]. According to the findings of relevant research, these mediators of inflammation and immune regulation caused increases in vascular permeability, stimulated local nociceptors, and promoted the release of other mediators of inflammation. In addition, antioxidants such as peroxiredoxin 2 were found to be down-regulated in both PE and PCOS in placental and other tissues [59]. The results of this research have advocated that oxidative stress stimulates androgen-producing steroidogenic enzymes, leading to the hyperandrogenism observed in women with PCOS. Likewise, given the essential role of peroxiredoxin in protecting cells against H_2_O_2_-induced cell damage and apoptosis, down-regulation of antioxidants in the placentas of women may result in oxidative stress, a potentially important factor in the development of PE. Spiral arteries in the pregnant uterus are remodeled from low-flow, high-impedance vessels into large-caliber vessels with high-flow and low-impedance. During pregnancy, fetal extravillous trophoblasts (EVT) accumulate around vessels and start remodeling of tissues via their cleaning action before invasion [60]. It is postulated that the increased risk of GH-PE is caused by enhanced EVT apoptosis [61]. These findings and explanations may provide a scope for the linkage of these two diseases, which possibly share common pathways.

In this study, metformin use for PCOS did not lower the risk of subsequent GH-PE, similar to the results of a previous report [48]. The mechanism of action for metformin involves the improvement of hyperinsulinemia, androgen excess and anovulation by enhancing insulin sensitivity [48]. However, the occurrence of GH-PE is multifactorial and complicated, and cannot be attributed to a single etiology. Therefore, the effect of metformin on reducing the risk of future GH-PE might not reach statistical significance. It is also possible that metformin is usually only used for women with more severe PCOS, as those with mild symptoms may not need medication. Thus, the benefit of using metformin to protect women from GH-PE may be offset by the deteriorative effect of PCOS of high severity that results in metformin use. An investigation of the potential mechanisms and factors is beyond the scope of the current study, and in the future, more effort can be done to explore the etiologies and medical treatment for hypertensive women with preceding PCOS.

The strengths of the study include a large sample size, sound sampling method, and stricter selection criteria for women diagnosed with PCOS, SLE, and GH-PE. A major advantage of the current study is that the sample was retrieved from the database of a general survey of a national population rather than from purposive sampling in other studies. Furthermore, the sample size of the current study is large in comparison to other similar studies investigating the prevalence of GH-PE for women with preceding PCOS or SLE. We have robust results because of minimized possible errors that result from the sampling process. Moreover, all diagnoses for women with PCOS, SLE or GH-PE were made by objective laboratory blood and urinary tests, ultrasonography and physical examinations rather than the subjective judgment of physicians, in comparison to the past studies [41,45]. All biases originating from the sample, case selection, and physicians were reduced by the criteria and methods used in the study.

There exist some inherent limitations despite several strengths of the study. The first limitation lies in that the real status of clinical conditions was not entirely reflected by use of ICD codes alone. In response to the issue of appropriateness of the case definition, not only ICD codes but also reports of ultrasound, laboratory tests and physical examinations of medical claims in the national database were identified as basic criteria for inclusion. Secondly, medical data were gathered for reimbursement under the NHI system. Therefore, medical data may have been inconsistently gathered, possibly affecting the results. In NHIRD, there was a lack of some personal characteristics and medication data, including marital status, social status, smoking habit, body mass index (BMI), and out-of-pocket expenses for instruments and drugs such as intrauterine devices and oral contraceptives. The effect of such factors or non-metformin treatments could not be investigated because the personal height, bodyweight, out-of-pocket expenses for instruments and drugs were not routinely recorded in NHIRD. Furthermore, even if the power of the subgroup analyses was adequate (≧1800 females), the information on metformin dosage and duration could not be obtained, which was an inherent limitation of NHIRD and could possibly affect the results of the study. Moreover, the study did not investigate the effect of ethnicity on PCOS, SLE, and GH-PE due to ethnic homogeneity in Taiwan. Therefore, the results might not be generalized to other ethnic populations. Finally, the study could not differentiate between early onset (<34 weeks) preeclampsia (EOP) and late onset (>34 weeks) preeclampsia (LOP). Certainly, it is interesting to explore if either prepregnancy SLE or PCOS has predominance over EOP or LOP, however this topic is beyond the scope of the current study and warrants for further investigation.

## 5. Conclusions

The nationwide population-based study demonstrates that pregnant women with prepregnancy PCOS or SLE have an approximately two-fold risk of developing GH-PE compared to those without prepregnancy PCOS or SLE. Metformin usage for PCOS does not lower the risk of subsequent GH-PE. Clinicians should realize the elevated risk of subsequent GH-PE among women with prepregnancy PCOS or SLE. Preparation and monitoring before and during delivery should be increased for affected women to avoid the serious complications of GH-PE. The current study has provided evidence-based information; however, many aspects regarding this issue remain unknown and warrant further investigation.

## Figures and Tables

**Table 1:** Comparisons of the characteristics between the exposure (prepregnancy PCOS) and contrast (no PCOS) groups.

	Exposure Group		Contrast Group		Statistics		
	Primiparas with prepregnancy PCOS		Primiparas without PCOS				
	(*n* = 1,953)		(*n* = 7,812)				
	N	%	N	%	OR	[95% CI]	*p*-value
Age at diagnosis (y/o)	27.49 ± 4.83		27.49 ± 4.83				1.0000
15–25	672	34.41	2688	34.41			
26–35	1186	60.73	4744	60.73			
36–45	95	4.86	380	4.86			
Occupation							<0.0001***
White collar	1223	62.62	4192	53.66			
Blue collar	321	16.44	1517	19.42			
Retired and other	409	20.94	2103	26.92			
Urbanization							0.0007**
Urban	1319	67.54	4928	63.08			
Suburban	510	26.11	2373	30.38			
Rural	124	6.35	511	6.54			
Economic status (insurable wage)							<0.0001***
≧40,000 NTD	443	22.68	1164	14.90			
20,000–40,000 NTD	833	42.65	3378	43.24			
<20,000 NTD	405	20.74	1891	24.21			
Retired and others	272	13.93	1379	17.65			
Comorbidities							
Diabetes mellitus	86	4.4	102	1.31	3.48	[2.60–4.66]	<0.0001***
Dyslipidemia	87	4.45	218	2.79	1.62	[1.26–2.09]	0.0002**
Ischemic heart disease	26	1.33	108	1.38	0.96	[0.62–1.48]	0.8619
Cerebrovascular disease	14	0.72	105	1.34	0.53	[0.30–0.93]	0.0238*
Chronic pulmonary disease	304	15.57	1190	15.23	1.03	[0.89–1.18]	0.7148

*p < 0.05, **p < 0.001, ***p < 0.0001, by chi-square test or student’s t-test, as appropriate; Data are expressed as the number (%) or mean ± standard deviation, as appropriate.

**Table 2:** Comparisons of the characteristics between the exposure (prepregnancy SLE) and contrast (no SLE) groups.

	Exposure Group		Contrast Group		Statistics		
	Primiparas with prepregnancy SLE		Primiparas without SLE				
	(*n* = 820)		(*n* = 3280)				
	N	%	N	%	OR	[95% CI]	*p*-value
Age at diagnosis (y/o)	29.51 ± 6.44		29.51 ± 6.44				1.0000
15–25	232	28.29	928	28.29			
26–35	430	52.44	1720	52.44			
36–45	158	19.27	632	19.27			
Occupation							0.4790
White collar	462	56.34	1772	54.02			
Blue collar	166	20.24	689	21.01			
Retired and other	192	23.41	819	24.97			
Urbanization							0.3930
Urban	534	65.12	2101	64.05			
Suburban	239	29.15	947	28.87			
Rural	47	5.73	232	7.07			
Economic status (insurable wage)							0.0146*
≧40,000 NTD	184	22.44	583	17.77			
20,000–40,000 NTD	344	41.95	1427	43.51			
<20,000 NTD	164	20.00	752	22.93			
Retired and others	128	15.61	518	15.79			
Comorbidities							
Diabetes mellitus	36	4.39	72	2.20	2.05	[1.36–3.08]	0.0004***
Dyslipidemia	52	6.34	121	3.69	1.77	[1.27–2.47]	0.0007***
Ischemic heart disease	26	3.17	49	1.49	2.16	[1.33–3.50]	0.0014**
Cerebrovascular disease	29	3.54	53	1.62	2.23	[1.41–3.53]	0.0004***
Chronic pulmonary disease	166	20.24	487	14.85	1.46	[1.20–1.77]	0.0002***

*p < 0.05, **p < 0.01, ***p < 0.001, by chi-square test or student’s t-test, as appropriate; Data are expressed as the number (%) or mean ± standard deviation, as appropriate.

**Table 3:** Risk analyses of subsequent GH-PE in primiparas with prepregnancy PCOS and SLE.

Group	No GH-PE		GH-PE		Statistics	
	N	%	N	%	Crude OR	Adjust OR
					[95% CI]	[95% CI]
Contrast group (no PCOS; *n* = 7,812)	7638	97.77	174	2.23	Reference	Reference
Exposure group (PCOS: *n* = 1,953)	1840	94.21	113	5.79	2.70^***^	2.36***
					[2.12–3.44]	[1.83–3.05]
Subgroup in exposure group						
No metformin use (n = 1,752)	1654	94.41	98	5.59	Reference	Reference
Metformin use (n = 201)	186	92.54	15	7.46	1.36	1.44
					[0.78–2.39]	[0.81–2.56]
Contrast group (no SLE; *n* = 3,280)	3221	98.20	59	1.80	Reference	Reference
Exposure group (SLE: *n* = 820)	792	96.59	28	3.41	1.93^**^	1.95**
					[1.22–3.05]	[1.23–3.10]

^**^p < 0.01, ^***^p < 0.001; Odds ratio (OR) and 95% confidence intervals [95% CI] are calculated by logistic regression analysis, as compared to the reference group; Adjusted for age at the time of initial diagnosis of PCOS and SLE, occupation, urbanization, economic status, comorbidities.
